# Urinary proteomics and metabolomics studies to monitor bladder health and urological diseases

**DOI:** 10.1186/s12894-016-0129-7

**Published:** 2016-03-22

**Authors:** Zhaohui Chen, Jayoung Kim

**Affiliations:** Advanced Clinical Biosystems Research Institute, Cedars-Sinai Medical Center, Los Angeles, CA USA; Department of Surgery, Cedars-Sinai Medical Center, 8700 Beverly Blvd, Los Angeles, CA 90048 USA; Department of Biomedical Sciences, Cedars-Sinai Medical Center, 8700 Beverly Blvd, Los Angeles, CA 90048 USA; Department of Medicine, University of California, Los Angeles, CA USA

**Keywords:** Urinary biomarkers, Proteomics, Metabolomics, Bladder diseases

## Abstract

**Background:**

Assays of molecular biomarkers in urine are non-invasive compared to other body fluids and can be easily repeated. Based on the hypothesis that the secreted markers from the diseased organs may locally release into the body fluid in the vicinity of the injury, urine-based assays have been considered beneficial to monitoring bladder health and urological diseases. The urine proteome is much less complex than the serum and tissues, but nevertheless can contain biomarkers for diagnosis and prognosis of diseases. The urine metabolome has a much higher number and concentration of low-molecular metabolites than the serum or tissues, with a far lower lipid concentration, yet informs directly about dietary and microbial metabolism.

**Discussion:**

We here discuss the use of mass spectrometry-based proteomics and metabolomics for urine biomarker assays, specifically with respect to the underlying mechanisms that trigger the pathological condition.

**Conclusion:**

Molecular biomarker profiles, based on proteomics and metabolomics studies, reliably distinguish patients from healthy controls, stratify sub-populations with respect to treatment options, and predict therapeutic response of patients with urological disease.

## Background

Personalized medicine aims for a customized healthcare for each patient to match treatments with the right patients at the perfect timing. Gene-specific data (SNP genotyping as well as epigenetics) is too static to enable such timed treatments. It is therefore essential to collect variable biomarker, along with other clinical information, data to achieve accurate diagnostic assessment for individual patients [1–3]. Multi-omic readouts of cellular and organ phenotypes (RNA-Seq, proteomics and metabolomics) will be indispensible in the era of personalized medicine. Only through a combination of exact genotypic and molecular phenotypic information we will improve the development of custom and precision therapies [4–6]. Sub-grouping of patients is necessary to define the evidence-based protocol for matching treatments to the right patients with appropriate timing [5, 7]. The necessity of compiling molecular information and clinical outcomes in personalized medicine prompted us to believe that the use of multi-omic data in conjunction with clinical outcome data is ever more important not only at the time of medical intervention, but throughout patients’ lives. The need for and possibilities associated with big data approach to gain insight into biological processes driving diseases and to identify novel diagnostics is enlarging. In this review, we will discuss how far metabolomic and proteomic approaches have come to aid in this long-term goal.

Urological diseases including urological cancers and benign bladder dysfunctions are complex in nature and require powerful, precise treatments. Tests to find patient candidates for a specific or combination of therapy and to identify biomarkers are incredibly challenging to determine [6, 8, 9]. Urine contains information not only from the urinary track, but also from other organs, providing biomarkers for bladder and other systemic diseases [10–12]. Looking at urine data in conjunction with other available patient clinical data may enable us to understand the molecular signature, which helps monitor the stages of the diseases and responses to therapies. This is particularly true in urological diseases, where urine samples provide the primary window for diagnosis and drug behavior observation [13].

A common definition of the proteome is the entire set of proteins expressed by a cell, tissue or organism at a certain time. Since proteomics is the large-scale study of proteome, it can contribute to expanding the understanding of biological systems and functions in cells or organs. Proteomes are directly responsible for cell functions, and therefore, abnormal protein expression is an indication of cellular disruption due to the pathological conditions [14, 15]. Current global proteomic technologies may provide a comprehensive understanding of urological diseases, characteristics of the disease’s state, and novel approaches to relieve the clinical symptoms [16–18].

Metabolomics provides a global chemical fingerprint of the metabolism of cells and indicates physiological and pathological states of biological samples [19–21]. Thus, the power of metabolomics opens up an unparalleled opportunity to query the molecular mechanisms of the disease. Metabolites are not merely the end products of gene/protein expression, rather, they are the result of the interactions of the genome and proteome with their environment in the cells. They play as powerful mediators of cellular events both in long-distance actions (e.g. hormones), stress and physiological actors (e.g. oxylipins) [22] and as cell-internal mediators (e.g. α-ketoglutarate in pluripotency) [23]. Thus, analyzing metabolic differences between pathological and normal conditions could provide undiscovered insights into the underlying disease pathology.

In addition to the advancements in multi-omics data acquisitions, novel bioinformatics methods enable an integrated view to identify the combined action of biomarkers as well as to develop drugs [24–27]. A significant volume of data with various omics data, including genetic, epigenetic, transcriptomic, proteomic, metabolomic and clinical outcome data, provides researchers with the capability to see a broader perspective and make discoveries that couldn’t previously be delivered [28–31]. Integrative approaches have become the essential part of experimental designs aimed at better understanding the biology of bladder diseases.

The main goal of this article is to provide the reader with an up-to-date summary of the main molecular variations taking place in biofluids with respect to various urological diseases including urological cancers (e.g., prostate cancer (hereafter PCa) and bladder cancer (BCa)) and benign bladder dysfunctions (e.g., benign prostatic hyperplasia (BPH), interstitial cystitis/pelvic bladder syndrome, bladder pain syndrome (IC)), as well as of the analytical strategies employed to unveil urinary biomarkers.

We here focus on mainly two omics analyses—proteomics and metabolomics—and associated data integration strategies. These approaches enable researchers to: (a) identify unknown molecular mechanisms; (b) select molecular markers that can be used for drug discovery, preclinical, and clinical drug development; (c) develop diagnostic tools. First, we present a short review on the urine-based studies. Second, we discuss analytical techniques that are used in urinary omics analyses, including computational methods for data processing. Next, we present studies that have used proteomics or metabolomics approaches to reveal the fingerprints of urological diseases. Finally, we discuss the future research directions and prospective how to apply to diagnosis and precision medicine for patients to summarize the review.

## Discussion

### Urine-based biomarkers for diagnosis, prognosis, or monitoring the treatment efficiency

A concerted effort bridging basic biology and clinical research is needed to identify high quality predictive biomarkers [31]. Discovery and validation of predictive biomarkers should be an integral part of clinical trials. In the clinical setting, the best diagnostic value is given by noninvasive biomarker tests that have both high sensitivity and specificity. A non- or minimally invasive diagnostic method using biofluids (e.g., urine, blood, saliva, fecal extract, and sputum specimens) may play a significant role in urological diseases with regard to early detection, diagnosis, prognosis, drug development, and sensitivity prediction to clinical treatments [12, 32–34].

So far the most attractive biofluids for biomarker discovery in bladder health and urological diseases are serum and urine [32–34]. Serum is a relatively accessible, stable and informative biofluid, making it ideal for early detection of systemic alteration in a wide range of diseases [35, 36]. Monitoring of serum has several advantages mainly due to its stability and minimum dilution effect. Proteomic and metabolic profiles of serum can be regarded as important indicators of physiological and pathological states and may aid in the understanding of the mechanism behind disease occurrence and progression [37–39]. However, blood samples pose certain disadvantages. During blood sample collection, proteases are often activated, which degrades proteins quickly and introduces a range of variability. On the other hand, 20 highly abundant proteins in the blood, which correspond to 99 % of the proteins, may hinder the identification of other less abundant, potentially important, proteins [40–43]. This feature makes it challenging to develop plasma or serum based assays and often analytes enrichment or protein depletion is needed.

Urine definitely is not a waste in regards to gaining patients’ diagnostics and therapeutic information [18, 44, 45]. However, it is still in debate whether urine plays an active role in regulating bladder biology. Urine’s composition is 95 % of water with small amounts of ammonia, sulfate, and other constituents. Total protein concentration in urine from healthy donor is very low (<100 mg/L) and urinary proteome contains over 100,000 different peptides [18, 32, 44, 46, 47]. Approximately 1500 proteins have been shown to constitute the urinary proteome, of which large proportions are extra cellular proteins, plasma membrane proteins, and lysosomal proteins [18, 48]. The Human Kidney and Urine Proteome Project by the Human Proteome Organization (HUPO) suggested that urine is an ultra filtration of the blood in the body, since urine and blood samples share the proteome profile [49–51]. Approximately 30 % of the proteins in normal human urine are plasma proteins, while the other 70 % are proteins derived from the kidney and genitourinary tract [49, 50].

Urine samples usually need special treatments to meet the requirements of reproducible measurements after sample collections. To obtain reliable and consistent profiles of urine, first, urine must be collected in a sterile bag or plastic container, because urinary bacteria metabolism significantly interferes on the urine proteome and metabolome. Secondly, urine samples must be properly processed (e.g., pH adjustment and/or removal of cell debris) and frozen at −80°C immediately after collection, until analysis [40, 46]. In addition, analysis of urine samples poses several analytical challenges for profiling owing to wide variations in the ionic strength, pH, and osmolality, particularly under conditions of physiological stress, diet, exercise, medication, health condition, and environmental exposure [46, 52, 53]. Furthermore, urine samples typically have a huge dynamic range of metabolite and protein concentrations. Another potential problem is the presence of proteolytic activity in the urine by urokinase and other enzymes [54]. Proteases found in stored urine degrade urinary albumin to a substantial degree. However, the extent to which proteases affect biomarkers in the urine is still unclear.

Despite all these shortcomings, urine is still an attractive source for studying bladder diseases. To monitor bladder condition, urine-based assays present the most attractive strategy, among other biofluids-based methods, given that the body fluids that are most proximal to a disease site often can provide a source of informative biomarkers. Urine is readily obtained and available with no required preparations by the patient and it is less complex than other body fluids. The ease of collection allows for serial sampling to monitor disease and therapeutic responses. Care must be taken in interpreting urine-based proteomics and metabolomics data. The main disadvantage of urine is the variation in protein concentration due to differences in fluid consumption during the day, which can be countered by normalizing with creatinine. However, although creatinine is the best possible internal standard for correcting urine volume effects, creatinine levels can vary due to dietary intake and pathological conditions. Computational approaches for data normalization methods can be applied to reduce artifacts due to sample variability using currently developed probabilistic quotient- and median-fold changes in normalization strategies [55].

### Analytical techniques and databases for urine-based omics for bladder diseases

**Fig. 1:**
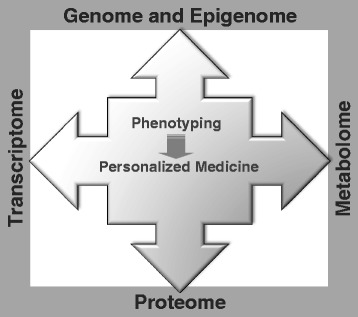
Overview of multi-omics technologies, which can be applied to urine-based biomarker study

**Fig. 2:**
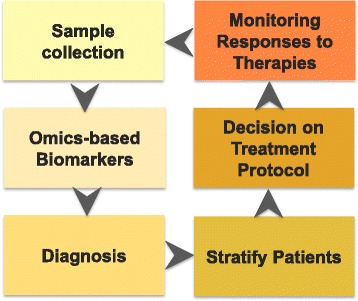
Potential clinical application using urine-based biomarkers

With the latest advances in high-throughput technologies, the pace of advances in the “omics” field accelerated the rate of novel biomarker discovery and therapeutic targets for various bladder diseases. Various omics technologies for personalized medicine are shown in Fig. 1, and ideal applications and workflow of urine-based biomarkers in clinical settings are shown in Fig. 2.

Proteomic technology has made a dramatic progress in the overall quality and information content over the past 5 years [56]. When computationally matching identified proteins (or metabolites) against knowledge-based databases, proteomics or metabolomics profiles today provide direct insights for biological interpretation of molecular perturbations unique in patients with urological diseases [47, 57, 58]. In this section, we review the current proteomic and metabolomic techniques and analytical tools/softwares that are used to identify signatures of urological diseases.

#### Urinary proteomics studies

Proteins are the major players influencing a person’s health, since proteins frequently have the greatest clinical significance for the diagnosis of diseases. Studies in the field of proteomics aim to elucidate proteomes and understand the identity, quantity, modification, localization, interaction, and function of all proteins in a given cell type or tissue. A number of powerful proteomic technologies were developed, demonstrating that proteomic approaches have wide utility [59, 60]. Proteomics profiling enabled the comparing of protein differences between patients suffering from a wide range of ailments and healthy controls to discover biomarkers for diagnosis and monitoring treatment response [49, 56]. Further developments to understand the post-translational modifications (PTMs) in tissues and biological fluids from patients have been achieved through the development of mass spectrometry instrumentation with increasing sensitivity [61, 62]. Established protocols for PTM enrichment and pipelines for high-throughput assays for clinical specimens may provide the potential of automated and large-scale identification and quantification of PTM-ome and its biological role in diseases [63].

For urine proteomics, many mass spectrometry techniques, such as 2D PAGE-mass spectrometry (MS), liquid chromatography-mass spectrometry (LC-MS/MS), capillary electrophoresis-mass spectrometry (CE-MS), surface-enhanced laser desorption/ionization time-of-flight mass spectrometry (SELDI-TOF MS), matrix assisted laser desorption/ionization time-of-flight (MALDI-TOF) MS and nano-liquid chromatography-tandem mass spectrometry (Nano-MALDI-MS) have been used with some advantages and limitations [64–68]. We described here only a few analytical tools that highlight the usefulness of it for urinary proteomics research. Briefly, 2D PAGE-MS is time consuming and technically challenging but very effective for large molecules. LC-MS is also time-consuming but pretty sensitive. CE-MS is cheap and good for biomarker discovery. MALDI-TOF MS is relatively simple, inexpensive, and, thus, a good option for fast screening. In general, nano-MALDI-MS is known to be much more sensitive than MALDI-TOF MS [64].

The gel-based 2-DE method enables urinary proteins to be resolved based on their molecular weight and isoelectric point. Several tools for image noise subtraction, protein spot detection, spot quantification, and spot matching can be used for 2-DE analysis including *Melanie*, *ImageMaster2D*, and *PDQuest* et al. The main steps in differential analysis of 2DE gels involve and statistical analysis. Often, the 2-DE method is coupled with MALDI-TOF MS or LC-MS/MS. Peptides from protein spots of interest are mixed with a matrix (*e.g.*, α-cyano-4-hydroxycinnamic acid) solution and are spotted onto a MALDI plate and analyzed with a MALDI-TOF MS to identify a peptide-mass fingerprint. These peptides can also be analyzed with nanoLC-MS/MS to sequence each peptide and thus identify the protein.

Besides identification and characterization, urine proteins can also be quantified. Today, label-free proteomics is the primary approach to relative quantifications of the human urinary proteome [69, 70]. A major advantage of label-free quantification is that this method is cheaper, simpler and involves less complicated data analysis than isotope-labeled approaches. Data processing is often performed by softwares such as *Decyder MS, Protein Lynx, SIEVE,* and *skyline* [71]. However, label-free quantification is limited by its lower quantification accuracy (especially for spectral counting in data dependent scan methods), and label-free data dependent acquisition quantifications are generally results in the identifications of less proteins and poor reproducibility. Currently SWATH and other data independent mass spectrometry acquisition methods and several computational algorithms are tested in their potential to overcome these limitations [59, 69, 70, 72].

The use of the most advanced proteomics mass spectrometry technologies has allowed discovering and verifying several urinary biomarkers of bladder diseases. In a large proteomics study, 407 patient urine samples were analyzed using MALDI-TOF MS. Two markers, uromodulin and semenogelin, could distinguish PCa versus BPH with 71.2 % sensitivity and 67.4 % specificity [9]. In another study on prostate cancer (PCa), capillary electrophoresis was coupled with MS detection of proteins and was able to identify and validate 12 novel urinary biomarkers for PCa [73]. This report suggested that collecting mid-stream urine samples was uninformative, but that first void urine was able to identify patients with PCa with 91 % sensitivity and 69 % specificity [73]. Due to its limited size, this study certainly requires additional validation in a larger cohort. In general, it can be assumed that a panel of biomarkers will most likely achieve an overall high level of specificity and robustness than using a single urinary protein biomarker. Further development of quantitative proteomics and selective or multiple reaction monitoring (SRM/MRM) methods [74–76] may allow the protein-quantification data to stand by their own without redundant validation using traditional protein quantification methods such as Western blot and ELISA. In many cases, there is no antibody available, and the capability of measuring multiple biomarkers in a panel for immune-based assays is very limited.

#### Urinary metabolomics studies

Metabolomic profiling, or metabolomics, is the systemic study of the unique small chemical fingerprints in a biological sample, and is the collection of small-molecule profiles that represent the end products of cellular processes in biological systems (e.g., cells, tissues, or organs) [20, 77]. As little as 5 ul of plasma or urine allows the characterization of hundreds of metabolites that provide a functional readout of the metabolic state. A recent effort to characterize the metabolomes of human urine has completed to identify and annotate approximately 2500 urinary metabolites using nuclear magnetic resonance spectroscopy (NMR, in most cases ^1^H-NMR), gas chromatography mass spectrometry (GC-MS), direct flow injection mass spectrometry (DFI/LC-MS/MS), inductively coupled plasma mass spectrometry (ICP-MS) and high performance liquid chromatography (HPLC) [78]. The detailed information of metabolite structures, concentrations, related literature references and disease associations is publically available via an online database (http://www.urinemetabolome.ca) [77]. Urinary metabolite levels are usually standardized by creatinine concentrations. Endogenous substrate levels in normal healthy subjects can inform on the status of each subject’s metabolizing enzyme activities. The comparison of urinary metabolite levels of patients vs. healthy controls, and responders vs. non-responders to a particular drug should facilitate the development of useful biomarkers to diagnose the disease or to predict the response, respectively. Also, understanding of urinary metabolome in healthy condition may help the titration of drug dose and monitoring drug response [18, 77].

Metabolomic studies typically begin with sample collection followed by sample analysis. A number of analytical techniques including NMR spectroscopy, GC-MS, and liquid chromatography-mass spectrometry (LC-MS) are used as methods of analysis [19]. NMR spectroscopy has proven to be particularly good for urine metabolomics analysis, because the technique is highly reproducible, requires minimal sample handling, and is straightforward to implement [79]. While the reproducibility, quantitative ability, and structure information derived from the NMR methods are big advantages, the relatively lower sensitivity and less straightforward identification methods are disadvantages of the NMR method [79]. MS-based metabolomics is considered more sensitive, providing greater coverage, and to be more cost-efficient than NMR-based applications. Given that the coverage varies with different technologies and instruments, the combination of different metabolomic approaches may provide a broad range of information that covers the metabolite profile and may maximize the capability of metabolomics analysis [19–21].

For metabolomics data processing, several statistical tools are currently used to analyze NMR and MS-based metabolomics datasets (e.g., MS-DIAL [80], XCMS, MZmine, MetAlign, MathDAMP, and LCMStats) [81, 82]. As metabolite databases, the Human Metabolome DataBase (HMDB), Madison Metabolomics Consortium Database, METLIN, and LipidMaps are generally used. To further understand the biology of the identified metabolites, HMDB (http://www.hmdb.ca/), METLIN (http://metlin.scripps.edu/), MassBank (http://www.massbank.jp), PubChem (https://pubchem.ncbi.nlm.nih.gov/) and KEGG (http://www.genome.jp/kegg/) can be used.

There is an increasing awareness of standardization or careful accounting in experimental design of urinary metabolomics study. To overcome possible limitations and pitfalls of the metabolomics approach, specific recommendations for urine collection, sample handling, storage, data acquisition, and statistical validation are also needed [78].

#### Urinary extracellular vesicle-derived omics studies

Most cells including cancer cells shed different types of vesicles into extracellular environment [83]. These vesicles are so-called extracellular vesicles (EV) including microvesicles, exosomes, and oncosomes, which are named based mainly on their size and characteristics [84]. EV have an increasing attention in the field of biomarker discovery. Given that EV are membrane bound structures, the components should be protected from degradation by extracellular proteases, DNAse and RNAse. A possibly selective package process during EV formation and shedding may lead to the reduced complexity of the contents [83, 84].

EV were originally considered a cleaning system to trash away the unnecessary molecules from cells. However, accumulated evidence demonstrates that EV influence their microenvironments by altering signaling pathways and delivering genetic information to other cells within close proximity [85–88]. Today, EV are accepted as potent mediators of cellular communication and as selectively packed delivery vehicle, which can provide clues to EV biogenesis, targeting, and cellular effects [87–89]. EV may also be used as a source of biomarkers for disease diagnosis, prognosis and response to treatment [89, 90]. Since EV can be readily isolated from multiple biological fluids (e.g., urine, serum, plasma, pleural effusion and saliva et al.), they have been considered to contain non-invasive biomarker candidates. In some pathological conditions including urological cancers, EV are easily secreted into the urine, and the urinary EV contain rich molecular information specific to the disease conditions such as cytoplasmic RNAs, miRNAs, metabolites and proteins [91]. Several disease-associated proteome were identified in urine from patients. Since EV-based urinary biomarkers are cell-free and do not rely on the presence of shed cells, urine provides a promise for the easy detection of bladder diseases [92, 93].

Unfortunately, there is no gold-standard technique for enriching and isolating EV in the clinical practice [94]. Nevertheless, several techniques have been developed to enrich and isolate urinary EV. This section discusses the different methods used to isolate urinary EV. Before isolating EV, it is advised to remove well-known abundant proteins in urine (e.g., uromodulin) [95]. Step-wise differential ultracentrifugation including low speed and high-speed centrifugation, and immuno-affinity and peptide-based isolation methods can be applied. The so-called Vn-96 peptide, based on surface marker of EV, was introduced to capture EV from biological fluids including urine. ExoQuick-TC™, Exospin™, and miRCURY™ EX isolation kits are based on aggregating agents followed by a low-speed centrifugation. Size-exclusion chromatography was also introduced to fractionate urine samples and isolate EV. Exochip™, a microfluidic-based method, has been recently shown to isolate EV. In particular, the hydrostatic dialysis method is efficient to enrich EV from highly diluted samples with molecular weight cut-off of 1000 kDa [94]. After omics analysis is done using EV isolated from urine samples, data can be analyzed using three major publically accessible EV-associated databases, EVpedia, ExoCarta, and Vesiclepedia [71, 96].

Because the variable results have been obtained with different isolation techniques, further discussion on the standard protocols for EV isolation, and normalization problem, which are major obstacles for the quantitative omics studies of EV, will be needed to apply this interesting biological resource into clinical practice.

#### Computational approaches to integrate data for better knowledge extraction

Using all information available from a wide variety of sources, including behavioral, genomic and life-style data has been coined “Big Data”. In clinical research, Big Data approaches show promise to connect information for individualized therapy approaches, called Personalized Medicine, once Big Data Initiative has been shown to lead to new scientific insights to better understand the biology [4]. Omics studies generate long lists of interconnected genes, proteins and metabolites, which may be integrated in clinical settings via computational approaches [18, 21, 28, 75]. The systems approach, integrating multi-omics, data will increase the reliability of discovering biomarkers and development therapeutic strategies for bladder diseases.

Currently available tools for integrating omics data can be categorized (i) to identify parameters of disease-associated biological networks and (ii) to identify pathway-based targets. Computational methods and tools for identification of important molecular targets and biomarker candidates are summarized. The major network-based visualization tools include VANTED (https://immersive-analytics.infotech.monash.edu/vanted/), VisAnt (visant.bu.edu/), Metscape2 (metscape.ncibi.org/metscape2/), Arena3D (arena3d.org/) and MetaMapR [97]. In order to construct a disease-perturbed network, several softwares and integrative querying systems for interaction information (PSICQUIC), network modeling and analysis tools (STRING [98] and Cytoscape [99]), and pathway analysis (KEGG [100]) might be useful. Commercial tools (e.g., GeneGo and Ingenuity Pathway Analysis (IPA)) are also helpful to construct a network. For pathway visualization, various tools are available, including Pathguide (www.pathguide.org/), KEGG-based pathway visualization tool (www.genome.jp/kegg/pathway.html), Paintomics (www.pantomics.com/), ProMeTra (https://www.cebitec.uni-bielefeld.de/polyomics/index.php/comics-software/75-prometra/), KaPPa-View (kpv.kazusa.or.jp/), MapMan (mapman.gabipd.org/), MAYDAY, and PaVESy (pavesy.mpimp-golm.mpg.de/). Based on the biochemical activities extracted from experimental datasets, interactive pathways can be constructed [101].

Importantly, in order to extract biological knowledge and to perform successful data integration across multiple resources, it is always essential to understand the context of the biology. Most current approaches, maybe with the exception of the Ingenuity Pathway analysis, are ignorant of disease etiologies and common pathological information that are very well known to clinical scientists. Hence, it is critical that scientists using pathway or genomic software are aware of this pitfall and use such network analyses only as additional tool to structure data and information, but not to expect immediate understanding. Only under careful interpretation of clinical knowledge and scientific literature can Omics data and software provide new hypotheses on undiscovered biological pathways and processes, eventually allowing us to personalized care and therapies on bladder diseases.

### Potential biomarkers of bladder diseases

**Fig. 3:**
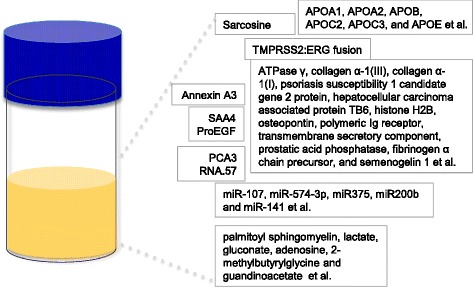
Examples of urine-based proteomic and metabolomic signatures of urological diseases

Next, we review the current state of proteomics and metabolomics in conjunction with recent technical advances in mass spectrometry in this section. The key applications and achievements by urinary proteomics and metabolomics in clinical biomarker research are discussed. Focus will be given to PCa, BCa, BPH and IC among other urological diseases. Examples of urine–based biomarkers suggested by previous studies are shown in Fig. 3.

#### Urinary biomarkers for prostate cancer

As the second most prevalent cancer in men, PCa’s incidence reaches 899,000 new cases and 258,000 deaths per year [102]. One of the gold standard diagnostic tools for PCa progression detection is the measurement of prostate specific antigen (PSA) in serum [102].

There have been many proteomic approaches to identify the urine-based biomarkers of PCa. For example, a large study using urine samples from 591 patients reported Annexin A3, a calcium-binding protein that plays a role in the regulation of cellular growth and in signal transduction pathways, as a novel urine-based biomarker for early PCa detection when used in conjunction with PSA [103]. Using CE-MS, 12 urinary biomarkers for PCa, including sodium/potassium-transporting ATPase γ, collagen α-1(III), collagen α-1(I), psoriasis susceptibility 1 candidate gene 2 protein, hepatocellular carcinoma associated protein TB6, histone H2B, osteopontin, polymeric Ig receptor, transmembrane secretory component, prostatic acid phosphatase, fibrinogen α chain precursor, and semenogelin 1, were identified and validated (91 % sensitivity and 69 % specificity) [104].

These findings strongly suggest that the use of a panel of biomarkers for disease diagnosis rather than a stand-alone biomarker, which may not be as specific, would benefit to diagnostic precision. However, unfortunately, currently none of these urinary protein biomarkers have been introduced into clinical practice, since current diagnostic biomarkers are suboptimal and of poor utility for low-grade disease and surveillance. To become routine tests, these biomarker candidates should be carefully tested in multicenter clinical trials and should be measured in biological fluids by robust, standardized analytical methods.

For development of metabolite markers, both LC-MS and GC-MS methods were applied to profile various clinical samples (including tissues, urine, and plasma) from PCa patients and identified 87 metabolites that distinguished PCa from normal subjects [105]. This study suggested that an interesting urinary metabolite, sarcosine (N-methylglycine), associates with PCa progression to metastasis with significant predictive value [105]. A following nested case control study showed that urinary sarcosine (and cysteine) levels were significantly higher in 54 PCa patients who had a recurrence after treatment [106]. However, another follow-up study done using an independent cohort of 106 PCa patients failed to reproduce the ability of urinary sarcosine (normalized to creatinine) as a PCa biomarker [107]. It is certainly possible that sarcosine may serve only as cell-internal signal, and not be excreted or shed into biofluids.

In addition, several cell-free and exosome-derived urinary microRNAs were suggested as PCa biomarkers [43]. The following reports provided evidence that circulating miRNAs might be a next-generation biomarker and contribute to cancer screening in non-invasive liquid biopsy. Only few studies for PCa-associated miRNA in urine were reported. Five of the miRNAs were differentially quantified in PCa patients compared to controls (miR-107, miR-574-3p, miR375, miR200b and miR-141) in urine of men with cancer, compared to that of healthy volunteers [108]. Among them, two miRNAs (miR-141 and miR-375) were also found higher in the PCa patient blood [108]. In the case of miR-141, the urinary levels were approximately 50 fold higher in metastatic PCa patients, compared to the healthy controls. Nilsson et al. found that exosomes were carriers for the TMPRSS2:ERG fusion, which is an early molecular event associated with PCa invasion, and PCA3 RNA.57, which were originally found as PCa biomarkers in prostate tissues [108].

Recently we also found interesting urinary miRNAs including virus-encoded miRNAs, which are specific to PCa, suggesting that this miRNA panel can be usable for the clinical setting [88]. This miRNA panel showed much better specificity and sensitivity to PSA for the early PCa patients whose serum PSA levels are undetectable [88]. In addition to RNA detection, proteomic profiling of exosomes and EV in human urine is underway and may lead to new biomarker development for a variety of diseases, including urological cancers and other benign diseases, with a hope of the potential use of EV as reservoirs of disease biomarkers.

#### Urinary biomarkers for bladder cancer

Urinary bladder cancer (BCa), the fourth most common cancer worldwide, is a significant cause of morbidity and mortality with a high recurrence rate [109]. For a follow-up surveillance, the diagnostic methods have been mostly instrumental in approaches including cystoscopy and cytology, which are painful and invasive. Thus, the molecular assays in a non-invasive fashion are needed for BCa patient surveillance at an early stage. High-throughput proteomic profiling technologies will identify molecular signatures that are associated with BCa, and will provide us understanding on bladder cancer biology, eventually leading to the development of targeted therapeutics [57, 110].

The complementary techniques of high-resolution MS and Western blotting/dot blot were able to quantify the urinary proteome specific to NMIBC. 29 proteins had a significantly higher abundance (*p* < 0.05) in urine samples of NMIBC compared with matched controls [111]. Another MS analysis using a Bruker Ultraflextreme MALDI-TOF-MS revealed that the urine peptidome was associated with MIBC [57, 112]. Using hexapeptide-based library beads and an antibody-based affinity column using the iTRAQ technique, six apolipoproteins (APOA1, APOA2, APOB, APOC2, APOC3, and APOE) were suggested as BCa-associated urine proteins [112]. In this study, SAA4 and ProEGF were also significantly altered in BCa subgroups [112]. The combined signatures of SAA4 and ProEGF were demonstrated to have a good diagnostic capacity (AUC = 0.80 and *p* < 0.001) on BCa [112]. The other urine proteomic study using 2-DE MS demonstrated the increased level of urinary apolipoprotein-A1 (Apo-A1) in BCa patients compared to control subjects. Additional validation assays (*n* = 379) supported that Apo-A1 could be used as a BCa biomarker with a sensitivity and specificity of 89.2 and 84.6 %, respectively [113].

An unbiased global metabolomic profiling using high-performance liquid chromatography-quadrupole time-of-flight mass spectrometry (HPLC-QTOFMS) profiled urine metabolites of BCa patients and controls. Comprehensive data analyses suggested 12 differential metabolites that contributed to the distinction between the BCa and control groups with a great sensitivity (91.3 %) and specificity (92.5 %) (AUC = 0.937) [114]. Interestingly, BCa-associated urinary metabolomes are enriched in glycolysis and beta-oxidation [114]. Recent urine metabolic profiling was performed on two subject cohorts with and without BCa in three independent platforms, which include ultrahigh-performance liquid chromatography/tandem mass spectrometry (UHPLC-MS/MS) in the negative ion mode, UHPLC-MS/MS in the positive ion mode, and GC-MS. As a set of candidate biomarkers for bladder cancer, 6 biomarkers (palmitoyl sphingomyelin, lactate, gluconate, adenosine, 2-methylbutyrylglycine and guandinoacetate) were suggested [115].

There is no study on urine exosome-derived miRNA signature associated with BCa, however, exosome proteomics studies demonstrated exosomes were highly purified from cultured BCa cells. Using ultracentrifugation on a sucrose cushion, Western blotting and flow cytometry of exosome-coated beads, 18 urine exosome proteins (e.g., basigin, galectin-3, and trophoblast glycoprotein (5 T4) et al.) were identified and validated [116], suggesting that exosomes in urine are a highly stable resource of biomarkers for BCa.

#### Urinary biomarkers for BPH

Incidence of benign prostatic hyperplasia (BPH), the most common benign disease among men, is known to be associated with age. Since BPH patients have similar symptoms to those of PCa patients, there have been diagnostic challenges in clinical settings.

The urine proteome-based method for discrimination of BPH from high-grade prostatic intraepithelial neoplasia or PCa was developed through testing 407 patient samples using MALDI-TOF [73]. Recently performed urinary proteome profiling of men with BPH vs. PCa using iTRAQ LC/LC/MS/MS have identified 25 proteins that were differentially expressed in urines [73]. Three proteins, β2M, PGA3, and MUC3, were further validated by western blot analysis. The combination of these three proteins showed an AUC of 0.710 (95 % CI: 0.631–0.788, *P* < 0.001) and enhanced a diagnostic accuracy when combined with PSA (AUC = 0.812, (95 % CI: 0.740–0.885, *P* < 0.001), suggesting a useful biomarker candidate panel segregating BPH from PCa [9].

#### Urinary biomarkers for IC

IC is a chronic bladder syndrome with bladder pain, urinary frequency/urgency, pressure, discomfort, and nocturia, which cause the suppressed physical function and social activity and adverse impact on the quality of life [117–119]. Approximately 1 out of 77 people in the United States have been diagnosed with IC. There is no gold standard for IC diagnosis. Objective diagnostic markers are urgently needed to improve prospects for clinical care. Etiologies of IC remain unknown. Prescription of medications has not been clearly suggested in clinical settings. Thus, there is a clear clinical need for the identification of biomarkers of IC.

The urine-based omics approaches to identify IC diagnostic markers have been employed. A small glycosylated peptide, antiproliferative factor (APF) was found in urine samples derived from IC patients [120]. Urinary APF bioactivity could segregate IC patients from controls (94 % sensitivity and 95 % specificity). The following global and unbiased quantitative proteomics combined with bioinformatics analysis performed by our group has enabled us to reveal the in vitro APF signaling network [121, 122]. Additional proteomics profiles associated with IC were suggested by studies using various technologies. Using 2-DE and MALDI-TOF, urine samples from 9 IC patients and 9 asymptomatic controls were analyzed, and the proteins such as uromodulin, kininogens (precursors of kinin) and inter-α-trypsin inhibitory heavy chain H4 were significantly altered in urine samples of IC patients [123]. A study by Kuromitsu et al. suggested that neutrophil elastase is significantly higher in IC subset with bladder pain and small bladder capacity than in other IC patients and healthy controls by using the 2D-DIGE nanoLC-MS/MS [124]. Another urinary proteome identified by Goo et al. revealed that α-1B-glycoprotein, orosomucoid-1, transthyretin and hemopexin were altered in 60 % of IC patients compared to controls [125].

A few attempts to use metabolomics analysis to identify an IC signature have suggested promising metabolite signatures specific to IC. Fukui et al. used ultra-performance liquid chromatography-mass spectrometry (UPLC-MS) and found that the urinary ratio of phenylacetylglutamine to creatinine can be correlated to the clinical grade of IC (e.g. mild to severe based on symptoms) [126]. A report from Van et al. has suggested that IC patients exhibited distinct MS and NMR spectral patterns from non-IC patients [127]. With follow-up studies in a larger cohort, global metabolite profiling combined with multivariate statistical and bioinformatics analysis may validated some of these compounds as important biomarker metabolites contributing to the biological responses, such as the drug-induced toxicity, or response as metabolic biomarkers.

## Conclusion: concluding remarks and perspectives

In this short review, we have provided information on the current state of ‘omics’ studies and available data sets relevant to bladder health and pathological condition, and presents opportunities for new research directed at understanding the pathogenesis of this complex condition. We believe that the ultimate goals of urine profiling of proteome and metabolome should be (i) to identify non-invasive diagnostic and prognostic biomarkers of bladder diseases, (ii) to better understand the biology of bladder diseases, and (iii) to determine the therapeutic strategies targeting the critical pathways of various bladder diseases. Recent efforts in the generation of large genomics, transcriptomics, proteomics, metabolomics, and other types of ‘omics’ data sets have provided a series of urinary biomarker candidates of bladder diseases. In spite of much efforts to identify candidate urinary biomarkers, it is still required to validate such markers in larger numbers of urine samples using targeted proteomics and metabolomics analyses in a prospective way.

Diagnostic and treatment modalities, even subjective diagnostic tools, are largely unavailable. As described here, our attempts to perform a systematic review and to build a pooled database using existing public ‘omics’ data associated with bladder health and various pathological conditions revealed the significant limitations and challenges facing investigators in the field. Many reports have suggested that natural diversity of patient population clearly plays a role in the difficulty of validating urine biomarkers. Expanding tests to include the general population often leads to loss or decrease in sensitivity. However, if tests are used for patients presenting specific symptoms in the clinic, and not for the general population, to inform about prognosis or treatment options, the pitfalls of general-population based urinary biomarkers may be alleviated. However, the cost of developing and validating a clinical grade assay is clearly beyond regular laboratory funding and would require concerted efforts by health agencies.

Collectively, despite these numerous pitfalls, urine is an interesting source of biomarkers for monitoring the bladder health. Rather than a single urinary molecular biomarker, a panel of biomarkers may be required to achieve the overall high level of specificity needed, so the trend is shifting towards implementing a panel of biomarkers, which may increase specificity. In order to translate potential biomarkers to clinical practice, vigorous validation must be pursued, with input from industry or large collaborative studies. Computational approaches combined with high quality ‘omics’ data could provide new insights in the field, essential molecular details about regulatory mechanisms and perturbations leading to bladder diseases, and essential information if we are to offer improved diagnostic capability and treatment strategies for patients.

## Availability of data and materials

All the data supporting your findings is contained within the manuscript.

## Abbreviations

APF: antiproliferative factor
BCa: bladder cancer
BPH: benign prostatic hyperplasia
DEPs: differentially expressed proteins
DFI/LC-MS/MS: direct flow injection mass spectrometry
EV: extracellular vesicles
GC-MS: gas chromatography mass spectrometry
HMDB: human metabolome database
HPLC: high performance liquid chromatography
IC: interstitial cystitis/pelvic bladder syndrome/bladder pain syndrome
ICP-MS: inductively coupled plasma mass spectrometry
iTRAQ: isobaric tags for relative and absolute quantitation
LC-MS: liquid chromatography-mass spectrometry
MALDI-TOF: matrix assisted laser desorption/ionization time-of-flight
MIBC: muscle invasive bladder cancer
MS: mass spectrometry
nano-MALDI-MS: nano-liquid chromatography-tandem mass spectrometry
NMIBC: non-muscle invasive bladder cancer
NMR: nuclear magnetic resonance spectroscopy
PCa: prostate cancer
PTMs: posttranslational modifications
SELDI-TOF: surface-enhanced laser desorption/ionization time-of-flight
SILAC: stable-isotope labeling by amino acids
SRM/MRM: selective or multiple Reaction monitoring
